# DNA Barcoding through Quaternary LDPC Codes

**DOI:** 10.1371/journal.pone.0140459

**Published:** 2015-10-22

**Authors:** Elizabeth Tapia, Flavio Spetale, Flavia Krsticevic, Laura Angelone, Pilar Bulacio

**Affiliations:** 1 CIFASIS-Conicet Institute, Rosario, Argentina; 2 Fac. de Cs. Exactas e Ingeniería, Universidad Nac. de Rosario, Rosario, Argentina; University Medicine Greifswald, GERMANY

## Abstract

For many parallel applications of Next-Generation Sequencing (NGS) technologies short barcodes able to accurately multiplex a large number of samples are demanded. To address these competitive requirements, the use of error-correcting codes is advised. Current barcoding systems are mostly built from short random error-correcting codes, a feature that strongly limits their multiplexing accuracy and experimental scalability. To overcome these problems on sequencing systems impaired by mismatch errors, the alternative use of binary BCH and pseudo-quaternary Hamming codes has been proposed. However, these codes either fail to provide a fine-scale with regard to size of barcodes (BCH) or have intrinsic poor error correcting abilities (Hamming). Here, the design of barcodes from shortened binary BCH codes and quaternary Low Density Parity Check (LDPC) codes is introduced. Simulation results show that although accurate barcoding systems of high multiplexing capacity can be obtained with any of these codes, using quaternary LDPC codes may be particularly advantageous due to the lower rates of read losses and undetected sample misidentification errors. Even at mismatch error rates of 10^−2^ per base, 24-nt LDPC barcodes can be used to multiplex roughly 2000 samples with a sample misidentification error rate in the order of 10^−9^ at the expense of a rate of read losses just in the order of 10^−6^.

## Introduction

Molecular barcoding provides the opportunity to multiplex next-generation sequencing [1] capacity across multiple individuals at specific portions of the genomes [2, 3]. As a result, cost-effective solutions able to accommodate a wide range of coverage demands can be accomplished [4]. Molecular barcoding lays on the ability of rather short oligos, known as barcodes, to tag DNA fragments belonging to different samples. Barcodes, which can be deployed either as part of adapters [5–7] or amplification primers [2, 4, 8], are expected to simultaneously offer negligible interference with DNA sequencing reactions, high resilience against sequencing errors and high multiplexing capacity.

Current barcoding systems are mostly designed with exhaustive methods. Large sets of random DNA sequences of size *N* are first screened to ensure the satisfiability of chemistry constraints imposed by the target sequencing technology, e.g., barcodes designed for pyrosequencing platforms must avoid homopolymer regions. Candidate barcodes are then screened to ensure a minimum pairwise distance *d*_*min*_ that guarantees the unambiguous correction of $\left[\frac{d_{min}-1}{2}\right]$ sequencing errors. The choice of the distance metric is determined by the type of sequencing errors. Pairwise Hamming distance evaluations of linear time complexity are required for mismatch sequencing errors. On the other hand, pairwise Levenshtein distance evaluations [9] of nearly-quadratic time complexity [10] are required for mismatch, insertion and deletion errors. In either case, a trade-off between *d*_*min*_ and the number *M* of legal barcodes must be accepted [11]. To overcome this problem, the straightforward use of larger random barcodes has been advocated. However, as *N* grows, exhaustive pairwise distance evaluations in search spaces of exponential growth are required. To simultaneously improve the multiplexing accuracy and the experimental scalability of random barcoding systems while keeping an acceptable computational complexity at the design time, combinatorial barcoding schemes have been proposed. In this regard, the paired-end-sequencing of hundreds of samples with few tens of barcodes tagging both ends of individual samples has been considered in [12–14]. However, although doubling the barcoding overhead roughly squares the multiplexing capacity of the initial set of barcodes and likely reduces multiplexation errors to some extent, the exact trade-off cannot be anticipated.

Demultiplexing of random barcodes relies on table-lookup decoding algorithms. For each received barcode, the closest legal barcode in a lookup table may be selected. Provided all barcodes are equally likely, such a decoding algorithm is a brute-force Maximum-Likehood (ML) decoder. A ML decoder minimizes the probability *p*_*e*_ of barcode identification error. For this purpose, a ML decoder always associates a legal barcode to a received barcode, although it may be other than the intended. Thus, ML decoding errors always go undetected, a feature that may seriously compromise barcoding applications requiring high specificity or equivalently, a strict control of the rate of false positives. Furthermore, since time complexity of ML decoding scales with the codebook size *M*, it should be only used in barcoding applications involving tens of barcodes built from a handy number of bases [15]. For more demanding barcoding applications involving tens of thousands of barcodes [16], ML decoding may be prohibitively time-consuming. Although several computational strategies may be used to alleviate ML decoding complexity of random barcodes, cumbersome data-dependent adjustments may be required [17]. Furthermore, for many important applications like the detection of rare mutations occurring at rates as low as 10^−8^ per base [18] or the counting of DNA/RNA templates [19, 20] at raw sequencing error rates of 10^−2^ per base [21–24], ML decoding may not be always the best choice: all decoding errors go undetected and result in samples misassignments.

To help in the fight against the rate of false positives in critical barcoding applications, undetected multiplexation errors must be controlled. For this purpose, incomplete decoders can be considered. For such decoders, *p*_*e*_ is split into the probability *p*_*u*_ of undetected multiplexation errors and *p*_*d*_, the probability of erasure decoding errors due to decoder failures, i.e., the decoder rejects to decide and data along the codeword, e.g., a sample identity, gets lost. Incomplete decoders can lower *p*_*u*_ at the expense of increasing *p*_*d*_. Hence, incomplete decoders allow us to exchange multiplexing accuracy by read losses, a feature that properly used can open the door to the design of highly accurate barcoding systems of overwhelming multiplexing capacity. A good example of this strategy can be found in the Illumina bcl2fastq demultiplexing software where only index reads with zero or one mismatch to a small reference index set are recovered. Although we expect that the *p*_*u*_ accomplished with the perfect match option is much lower than that with the one mismatch, we also expect that the corresponding *p*_*d*_ is much higher. Note that since *p*_*d*_ measures the expected rate of read losses, its behavior must be carefully monitored, especially for ultra-high-throughput sequencing systems where more stringent *p*_*d*_ requirements are necessary.

The DNA barcoding problem is indeed an instance of a largely studied problem in Communication Theory, the error-free transmission of discrete patterns in the presence of random noise [25], a problem which leads to the theory of error correcting codes. Since the recognition of this fact in 2008 [8], few works [26, 27] have considered the *systematic* design of coding-based barcoding systems, perhaps owing to the inherent difficulties of dealing with a problem which falls at the intersection between two quite different fields, Communication Theory and Molecular Biology.

In this paper, we attempt one step at bridging the gap, showing how state of art linear error correcting codes can be used for the systematic design of DNA barcodes able to accurately sustain the experimental scalability of current and upcoming sequencing technologies [28]. With main focus on sequencing systems impaired by mismatch errors, we generalize the design of BCH barcodes [26] by introducing shortened BCH barcodes, a class of barcodes built from binary BCH codes allowing otherwise prohibited barcoding sizes. To improve the design flexibility accomplished with shortened BCH barcodes, we further introduce LDPC barcodes, a class of barcodes built from quaternary LDPC codes [29]. Aiming to overcome the problem of undesirable homopolymer regions [11, 30] that likely reduces barcodes multiplexing capacity, and by the way to satisfy the key independence assumption between sequencing errors of BCH and LDPC decoding algorithms, the use of interleavers [31] is introduced. Simulation results show that using these design guidelines, highly accurate barcoding systems of high multiplexing capacity can be obtained with both BCH or LDPC codes. However, owing to their lower rates of read losses, LDPC barcodes may be particularly well suited for ultra-high-throughput sequencing systems.

## Results

Multiplexing capacity of barcoding systems is hampered by sequencing errors. Error correcting codes provide forms for redundant information representation and thus, the opportunity to correct random errors with high probability. Let us assume barcodes in *GF*(4) and some one-to-one mapping between field elements {0, 1, 2, 3} and each of the four DNA bases. To uniquely tag *M* samples, at least *k* = ⌈*log*_4_ *M*⌉ bases are needed and thus, if *n* > *k* bases are used, the *m* = *n* − *k* bases in excess can be used for error correction purposes. Sequencing errors can be broadly categorized into insertion, deletion, mismatch or substitution and erasure or ambiguous base-call errors. It is well-known that Roche/454 pyrosequencing platforms are prone to insertion and deletion errors over mismatch ones [32, 33] while Illumina reversible dye terminator chemistry platforms are definitely prone to mismatch errors over insertion and deletion ones; erasure errors, i.e., ambiguous base calls, are present in both platforms. In this paper, the design of barcodes for high-throughput sequencing systems mainly impaired by mismatch errors is considered.

### On the design of coding-based barcodes in GF(q)

Although sequencing errors occur in GF(4) [34], the systematic design of barcodes has been mostly confined to GF(2), the mathematical field where most successful Communication Theory results have been developed. This can be observed in recent proposals for the construction of barcodes from well-known binary linear codes equipped with algebraic decoding algorithms, e.g., Hamming, BCH and Golay codes [26, 35, 36]. Algebraic decoding of binary linear codes allows the correction of at least *t* ≥ 1 binary errors per corrupted codeword. By using one-one mappings between binary tuples {00, 01, 10, 11} and the four DNA bases, binary codewords can be mapped into candidate barcodes and thus, the correction of at least *b* mismatches in GF(4) can be mapped into the correction of at least *t* = 2*b* binary errors in GF(2).

Binary Hamming codes of size *n* = 2^*m*^ − 1 with *m* ≥ 4 able to carry *k* = *n* − *m* informative bits can be used to construct 2^k^ candidate barcodes of size *N* = (*n* + 1)/2. As *m* is increased, remarkable high multiplexing levels can be achieved with Hamming barcodes [8]. However, since *t* = 1 holds for all binary Hamming codes, Hamming barcodes cannot guarantee the correction of even *b* = 1 mismatches. To overcome this problem, barcodes built from quaternary extensions of binary Hamming codes have been proposed [27]. Note, however, that these barcodes, called BY in [37], do not conform to truly quaternary Hamming codes ([38] p. 55) and thus, their actual barcoding performance cannot be formally anticipated.

On the other hand, binary BCH codes of size *n* = 2^*m*^ − 1 with *m* ≥ 4 can be used for the construction of barcodes of *N* = 8, 16, 32… bases [26]. Since for a fixed code size *n*, multiple *t* > 1 options are possible, BCH barcodes can be used for the correction of at least $b\;=\;\left\lfloor \frac{t}{2}\right\rfloor$ base mismatches. However, since for a fixed code size *n*, increasing *t* lowers *k*, increased error correction power of BCH barcodes can only be accomplished at the expense of diminished multiplexing capacity.

To improve the design flexibility of BCH barcodes allowing intermediate *N* settings, shortened binary BCH codes can be considered. Shortening BCH codes with parameter *s* > 0 reduces the number of informative bits from *k* to *k*′ = *k* − *s* preserving the number of redundant bits. By means of shortening, BCH barcodes of size $N=\frac{n+1-s}{2}$ for *s* even or $N=\frac{n-s}{2}$ for *s* odd can be designed. To recover from sequencing errors, shortened BCH barcodes must be first demapped to the binary domain where earlier removed bits must be reinserted. Although shortening improves the design flexibility of BCH barcodes by permitting otherwise prohibited *N* settings, it does not allow arbitrary *k*′ and *t* settings and thus, suboptimal barcoding systems may be still obtained with shortened BCH codes. Beyond binary BCH codes, the famous binary extended Golay code [39] of size *n* = 24 able to carry *k* = 12 informative bits and to correct at least *t* = 3 binary errors can be also considered. Extended binary Golay codes can be used for the construction of barcodes of size *N* = 12 able to correct at least *b* = 1 base mismatches.

Recent years have witnessed a significant progress in the field of coding theory. This progress has been mainly boosted by the (re) discovery of binary LDPC codes [40, 41], a class of capacity approaching codes allowing an easy generalization to higher order fields [42], e.g., GF(4). LDPC codes are distinguished by their ability to exploit the statistic of symbol errors in a remarkable efficient way. As mentioned in [29], “it should be pointed out that all the errors were *detected* errors: the (LDPC) decoder reported that it had failed”, i.e., LDPC codes could be good candidates for the systematic design of highly accurate barcoding systems of high multiplexing capacity. Briefly, LDPC codes are linear block codes built from sparse pseudo-random bipartite graphs allowing a divide and conquer interpretation of the coding-decoding problem. The biggest difference between LDPC and both BCH and Golay codes is the way they are decoded. While binary BCH and Golay codes are decoded by algebraic methods, LDPC codes are iteratively decoded using their bipartite graph representation and the statistic of symbol errors, e.g., the mismatch error rate of sequencing machines. Note, however, that while long LDPC codes involving thousands of symbols are required for standard communication applications, short LDPC codes involving at most tens of symbols are required for DNA barcoding applications. As a result, an adaptation of well-established methods for the construction of good long LDPC codes is required. Taking into account that good short LDPC codes should resemble random counterparts [43, 44], a novel scoring system for the identification of quaternary LDPC codes with highly diverse parity check matrices was designed.

#### Good short LDPC codes for DNA barcoding applications

Parity check matrices for LDPC codes can be designed by random or structured methods. In the former case, the position and value of non-zero entries are determined by computer search. In the latter case, combinatorial methods over special classes of mother matrices are used. While structured methods are well-suited for constructing LDPC codes of large and moderate length, random methods are preferred for constructing short ones. Since LDPC codes required in the DNA barcoding framework are definitely short, random construction methods were used.

To minimize the impact of cycles at the iterative decoding stage, the positions of non-zero entries in quaternary LDPC matrices with *m* rows, *n* columns and *j* = 3 non-zero entries per column were first optimized with the Progressive Edge Algorithm (PEG) [45]. Resulting binary matrices were then used as templates for the generation of quaternary LDPC matrices by filling non-zero entries with elements carefully chosen from the set {1, 2, 3}. Regarding this important design issue, main focus of research has been put on the design of non-binary LDPC codes for binary communication channels [46]. In this regard, Mackay [47] proposed selecting non-zero entries to approximate an optimal decoder by maximizing the marginal entropy of parity check variables; under the assumption of a binary communication channel of the symmetric type, decoding improvements over the random assignment approach were observed. Similarly, Poulliat at al. [48] proposed selecting non-zero entries of non-binary LDPC codes based on the algebraic properties of their binary image representations.

We note, however, that the design criteria of quaternary LDPC codes for binary communication channels might not be applicable for quaternary ones. For example, for equiprobable quaternary errors like those assumed in our DNA barcoding framework, the marginal entropy of parity check variables of regular quaternary LDPC codes turns to be invariant to any selection of non-zero entries performed with the MacKay method. Since LDPC codes required for DNA barcoding applications are natively quaternary, and, so are the ideal equiprobable sequencing errors, alternative design approaches are required.

A novel score *D* designed to capture quaternary LDPC matrices *H* with the highest diversity between columns and between rows was devised. Regarding diversity between columns, we note that the minimum Hamming distance (*d*_*min*_) of a linear code equals the smallest number of linearly-dependent columns in **H** ([49] p. 13). Hence, a simple way to maximize *d*_*min*_ is to maximize the number of independent columns in **H**, e.g., by maximizing the number of distinct columns. Regarding diversity between rows, we built upon the optimization idea of Poulliat [48] that by maximizing the coding diversity between component parity check sub-codes defined by each **H** row, the more distinguishable the messages passed from check nodes to variable nodes will be so that improved iterative decoding performance should be expected.

An insight onto the diversity of **H** columns can be obtained from the vector of normalized pairwise Hamming distances between columns. This vector has size $\frac{n\cdot \left(n-1\right)}{2}$ and can be characterized by its mean *μ*_*h*,*c*_ and standard deviation *σ*_*h*,*c*_: we desire **H** matrices with the highest *μ*_*h*,*c*_ and the lowest *σ*_*h*,*c*_. Similarly, an insight onto the diversity of **H** rows can be obtained from the vector of pairwise cosine dissimilarity between rows. This vector has size $\frac{m\cdot \left(m-1\right)}{2}$ and can be characterized by its mean *μ*_*d*,*r*_ and standard deviation *σ*_*d*,*r*_: we desire **H** matrices with the highest *μ*_*d*,*r*_ and the lowest *σ*_*d*,*r*_. Hence, **H** matrices were scored as follows:

$$\begin{array}{c} D(H)=(\mu_{h,c}-\sigma_{h,c})\times (\mu_{d,r}-\sigma_{d,r}) \end{array}$$ (1)

In practice, multiple random quaternary parity check matrices **H** were generated from binary PEG templates and ranked with the *D*-scoring system. The best *D*-scoring **H** matrix was then selected for the generation of the corresponding LDPC barcoding system.

#### Interleaved coding-based barcodes

Naive elimination of barcodes with undesirable homopolymer regions [11] reduces the multiplexing capacity of general barcoding systems. To alleviate this problem in the design of BCH barcodes, the use of optimal position dependent mappings between binary tuples and quads in GF(4) has been proposed in [26]. Note, however, that such mappings may be difficult to obtain even for barcodes of modest size. To overcome this problem, the alternative use of interleaved coding-based barcodes is proposed. Hence, candidate barcodes coming from either binary BCH or 4-ary LDPC codes are first passed through an interleaver module [50] where undesirable homopolymer regions are hopefully broken. An interleaver simply permutes symbols from an input sequence according to a mapping. Interleavers can be constructed by pseudorandom or deterministic methods. Pseudorandom methods require to store the interleaving pattern in tables, which might be a problem for long barcodes. Since our barcodes are definitely short, interleavers were constructed with the semirandom permutation method described in [51]. Interleaved barcodes must be deinterleaved before their demultiplexation. By the way, deinterleaving helps to satisfy the key independence assumption between symbol errors required by standard decoding algorithms of BCH and LDPC codes. Since this assumption may be difficult to satisfy in current sequencing systems, interleavers provide a simple way to randomize otherwise correlated sequencing errors.

Besides limiting homopolymer regions and observing the independence assumption between symbol errors, the design of coding-based barcodes must also take into account well-known chemistry constraints, e.g., the *G* + *C* content and possible interference of barcodes with primer sequences. Most of these constraints have been already taken into account in the design of Barcrawl [52], a tool for the *ab-initio* design of primer barcodes for pyrosequencing applications. Hence, before their deployment, candidate barcodes are passed through an adapted version of the Barcrawl tool. In the modified Barcrawl version, the *ab-initio* generation of primer barcodes is suppressed and candidate barcodes are taken from interleavers output.

### DNA barcoding over mismatch sequencing channels

Barcoding systems built from binary BCH, binary Golay, quaternary LDPC and BY barcodes were evaluated using a Quaternary Symmetric Channel (QSC) model [53]. Under the QSC model, the *i*−*th* barcode symbol is ideally mutated from base *a* to base *b* with probability $p_{i}\left(b,a\right)=\frac{p_{s}}{3}$ for *a* ≠ *b* and remains unchanged with probability *p*_*i*_(*a*, *a*) = 1−*p*_*s*_. Following [54, 55], *p*_*s*_ ∈ [0.010, 0.075] was considered.

For practical purposes, *N* was limited to 25 bases. For each barcoding system of size *N* built with an error correcting code of size *n*, a wide range of error correction and multiplexing abilities were evaluated. This was accomplished by varying parameter *t* of binary BCH codes and parameter *m* of quaternary LDPC codes. For BCH barcodes, binary BCH codes of size *n* ∈ {15, 31, 63} and shortened versions of them were considered. For LDPC barcodes, quaternary LDPC codes of size *n* ≥ 16 were considered. LDPC codes of size *n* < 16 were disregarded due to difficulties in satisfying the mandatory LDPC sparse constraint. In addition, BY barcodes of size *N* ∈ {7, 8, 15} and Golay barcodes of size *N* = 12 were considered. For the sake of completeness, random barcodes reported in [56] of size *N* ∈ {8, 9, 10} and minimum edit (Levenshtein) distance *d*_*L*_ ∈ {3, 5, 7} were also considered. Recalling that the Hamming distance is an upper bound of the edit distance, random barcodes were further screened to determine their minimum Hamming distance *d*_*H*_. Similar values were observed, i.e., *d*_*H*_ ∈ {3, 5, 7} so that the correction of at least *t* ∈ {1, 2, 3} mismatch sequencing errors can be guaranteed.

Barcoding systems were evaluated through their multiplexing capacity *M*, their barcoding rate *B*, their probabilities *p*_*e*_ of barcode identification errors and their probabilities *p*_*u*_ of undetected multiplexation errors. For each *N*, *M* was defined as the maximum number of barcodes which were compatible with the given sequencing chemistry. Similarly, *B* was defined as the actual fraction of informative quads per barcode, i.e., $B=\frac{log_{4}M}{N}$; we expect *B* is close as possible to $r=\frac{k}{n}$, the chemistry unconstrained coding rate of underlying error correcting codes.

#### BCH, LDPC, BY, Golay and Random barcodes

Let us consider an ideal sequencing channel of the *QSC* type that generates mismatch sequencing errors with a probability *p*_*s*_. For a given set of sequencing chemistry constraints, the *M* and *B* accomplished by BCH and LDPC barcodes of size *N* will depend on the desired *p*_*e*_ and *p*_*u*_ for the given *p*_*s*_. With main focus on boosting experimental scalability without compromising multiplexation accuracy, BCH and LDPC barcodes of size *N* ≤ 25 able to fulfill the operational constraint *M* > ≥ 24 and *p*_*u*_ ≤ 10^−8^ at *p*_*s*_ = 10^−2^ were identified. For BCH barcodes, simulation results showed that the desired operational constraint could be only satisfied by shortening binary BCH codes of size *n* = 63. For *LDPC* barcodes, the desired operational constraint could be only satisfied by LDPC barcodes of size *N* ≥ 19.

As shown in Table 1, BCH barcodes of size *N* = 21 can be used to multiplex up to *M* = 86 samples with *p*_*e*_ ≈ 10^−5^ and *p*_*u*_ ≈ 10^−8^. By letting *N* to increase up to 25, one additional satisfactory configuration with *p*_*u*_ ≈ 10^−8^ can be identified at *N* = 22 with *M* = 384 and *p*_*e*_ ≈ 10^−5^. Note that for *p*_*u*_ ≈ 0, *p*_*e*_ essentially bounds the probability of read losses. Taking into account that the number of Illumina reads per flow cell currently ranges from 25 × 10^6^ to 300 × 10^9^, we may be interested in further *p*_*e*_ reductions.

**Table 1:** The performance of BCH barcodes.

				*p*_*s*_ = 10^−2^			
N	M	B	(n, k, t, s)	*p*_*e*_	$p_{e}^{+}$	*p*_*u*_	$p_{u}^{+}$
21	86	0.153	(63, 30, 6, 21)	6.94 10^−6^	6.99 10^−6^	1.00 10^−8^	1.02 10^−8^
22	384	0.195	(63, 30, 6, 19)	8.33 10^−6^	8.34 10^−6^	1.00 10^−8^	1.02 10^−8^
24	73	0.128	(63, 24, 7, 15)	1.84 10^−6^	1.85 10^−6^	0	2.00 10^−9^
25	295	0.165	(63, 24, 7, 13)	2.68 10^−6^	2.69 10^−6^	0	2.00 10^−9^

BCH barcodes of size *N* ≤ 25 constrained to accomplish *M* ≥ 24 and *p*_*u*_ ≤ 10^−8^ over a *QSC* model where mismatch errors occur with probability *p*_*s*_ = 10^−2^. *M*, *B*, *p*_*e*_ and *p*_*u*_ are respectively the empirical estimates of the multiplexing capacity, the barcoding rate, the probability of barcodes identification error and the probability of undetected multiplexing errors; $p_{e}^{+}$ and $p_{u}^{+}$ are the upper error bars of the two latter ones. Underlying codes are binary BCH codes of size *n* shortened to *n* − *s* able to carry *k* − *s* informative bits and to correct at least *t* binary errors.

**Fig 1:**
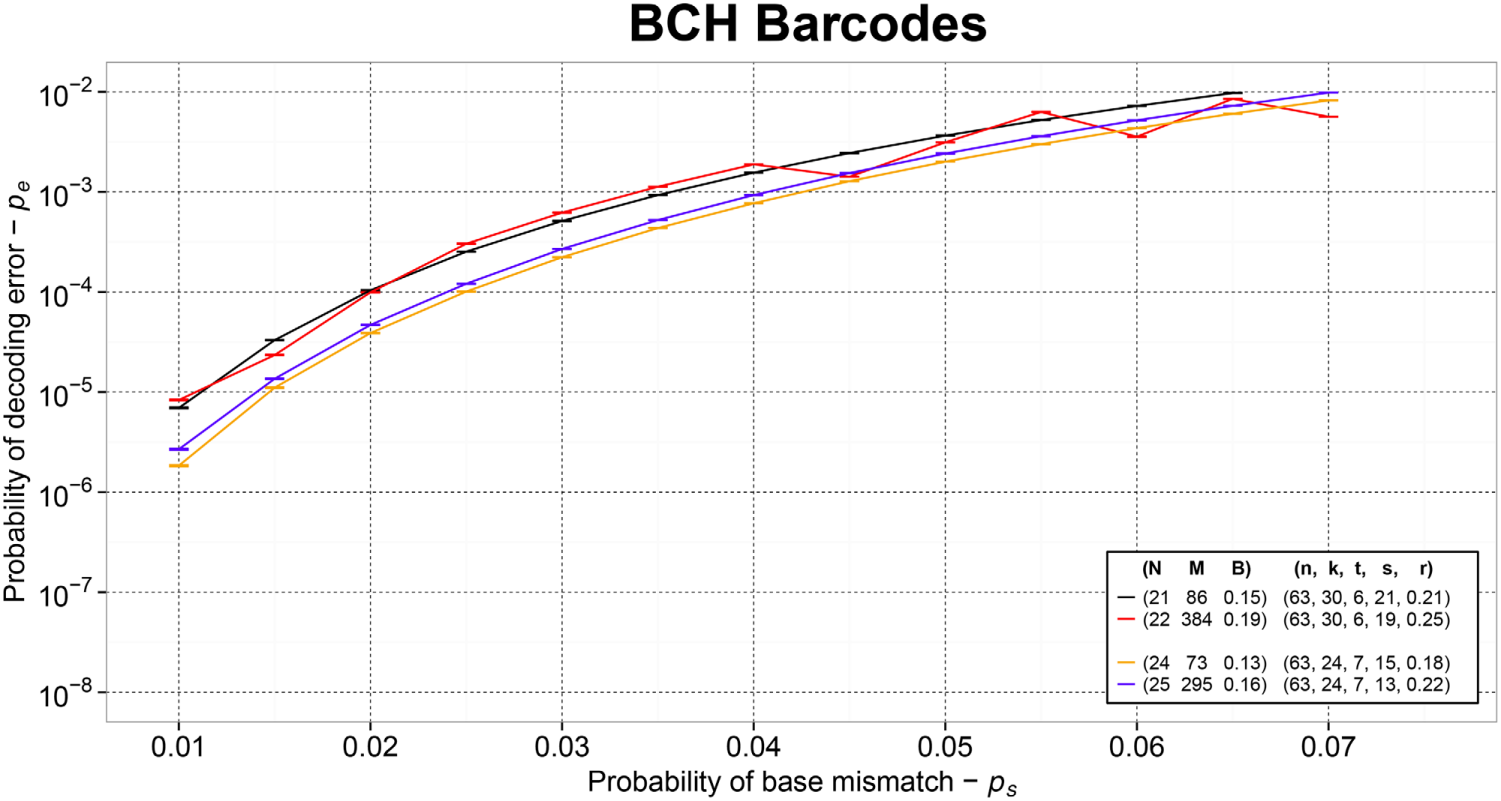
The empirical probability *p*_*e*_ of decoding error accomplished by BCH barcodes of size *N*, multiplexing capacity *M* and barcoding rate *B*. Sequencing errors follow a *QSC* model with probability *p*_*s*_. Binary BCH codes of size *n* shortened with parameter *s* able induce 2^*k*−*s*^ candidate barcode sequences and to correct at least *t* binary errors at a coding rate *r* are used.

Simulation results showed that to accomplish *p*_*e*_ ≈ 10^−6^, shortened BCH barcodes of size *N* at least 24 are required. BCH barcodes of size *N* = 24 can be used to multiplex up to *M* = 73 samples with *p*_*e*_ ≈ 10^−6^ and *p*_*u*_ ≈ 10^−9^. By letting *N* = 25, one additional satisfactory configuration with *p*_*u*_ ≈ 10^−9^, *M* = 295 and *p*_*e*_ ≈ 10^−6^ can be obtained. Details about the *p*_*e*_ performance of BCH barcodes beyond *p*_*s*_ = 0.01 are shown in Fig 1.

**Fig 2:**
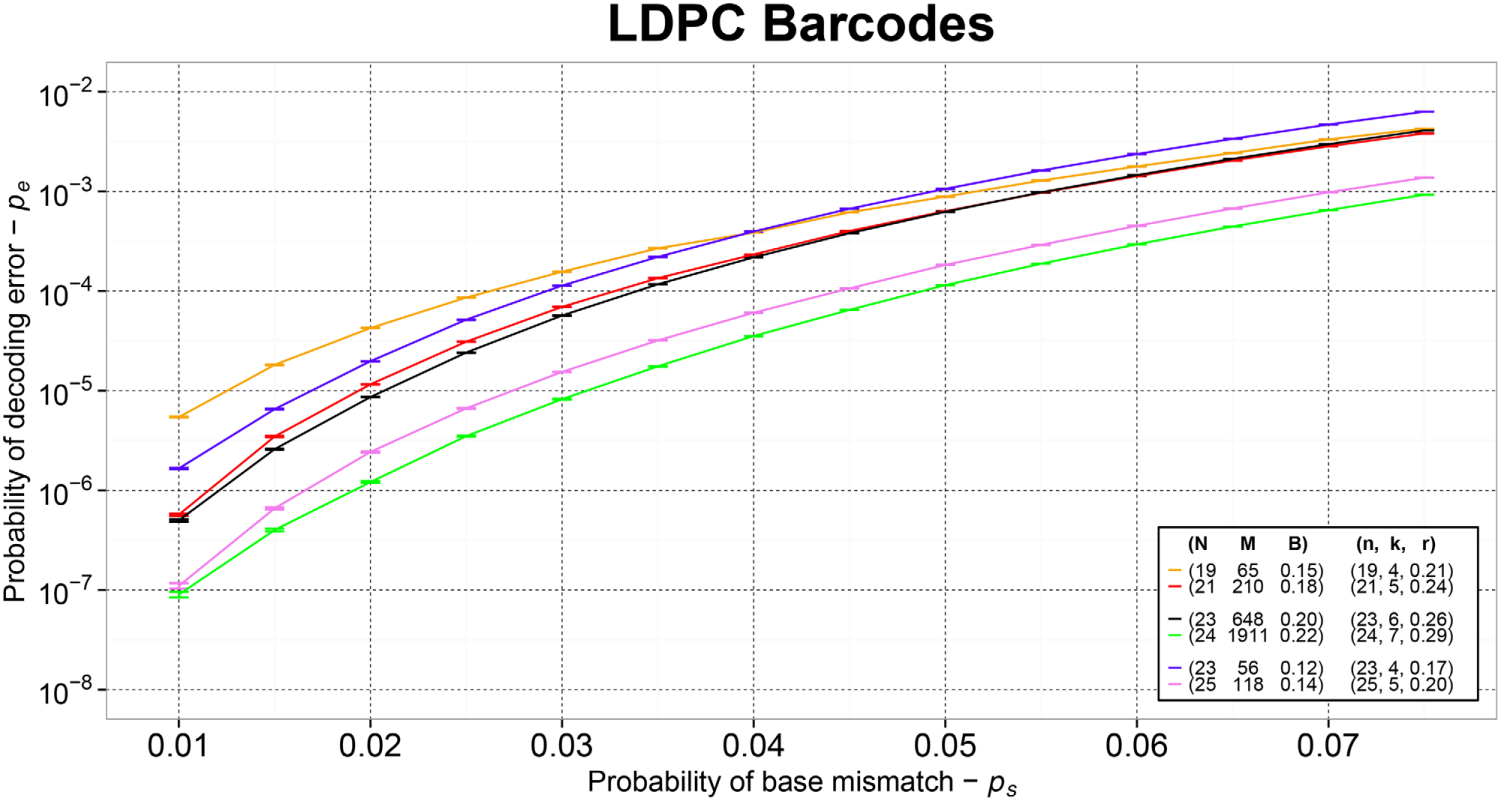
The empirical probability *p*_*e*_ of decoding error accomplished by LDPC barcodes of size *N*, multiplexing capacity *M* and barcoding rate *B*. Sequencing errors follow a *QSC* model with probability *p*_*s*_. Quaternary LDPC codes of size *n* = *N* able induce 4^k^ candidate barcode sequences at a coding rate *r* are used.

As shown in Table 2, LDPC barcodes of size *N* = 19 can be used to multiplex up to *M* = 65 samples with *p*_*e*_ ≈ 10^−5^ and *p*_*u*_ ≈ 10^−9^. By letting *N* to increase up to 25, three additional satisfactory configurations with *p*_*u*_ ≈ 10^−9^ can be identified at *N* = 21, 23, 24 with *M* = 210, 648, 1911 and *p*_*e*_ ≈ 10^−6^. To further reduce *p*_*e*_ in one order, LDPC barcodes of size *N* ≥ 23 are required. LDPC barcodes of size *N* = 23 can be used to multiplex up to 56 samples with *p*_*e*_ ≈ 10^−7^ and *p*_*u*_ ≈ 10^−9^. By letting *N* = 25, one additional satisfactory configuration with *p*_*u*_ ≈ 10^−9^, *M* = 118 and *p*_*e*_ ≈ 10^−7^ can be obtained. Details about the *p*_*e*_ performance of LDPC barcodes beyond *p*_*s*_ = 0.01 are shown in Fig 2.

**Table 2:** The performance of LDPC barcodes.

				*p*_*s*_ = 10^−2^			
N	M	B	(n, k)	*p*_*e*_	$p_{e}^{+}$	*p*_*u*_	$p_{u}^{+}$
19	65	0.158	(19, 4)	5.43 10^−6^	5.44 10^−6^	0	2.00 10^−9^
21	210	0.183	(21, 5)	5.70 10^−7^	5.72 10^−7^	0	2.00 10^−9^
23	648	0.203	(23, 6)	5.10 10^−7^	5.11 10^−7^	0	2.00 10^−9^
24	1911	0.227	(24, 7)	1.66 10^−6^	1.67 10^−6^	0	2.00 10^−9^
23	56	0.126	(23, 4)	9.10 10^−8^	9.11 10^−8^	0	2.00 10^−9^
25	118	0.137	(25, 5)	1.10 10^−7^	1.11 10^−7^	0	2.00 10^−9^

LDPC barcodes of size *N* ≤ 25 constrained to accomplish *M* ≥ 24 and *p*_*u*_ ≤ 10^−8^ over a *QSC* model where mismatch errors occur with probability *p*_*s*_ = 10^−2^. *M*, *B*, *p*_*e*_ and *p*_*u*_ are respectively the empirical estimates of the multiplexing capacity, the barcoding rate, the probability of barcodes identification error and the probability of undetected multiplexing errors; $p_{e}^{+}$ and $p_{u}^{+}$ are the upper error bars of the two latter ones. Underlying codes are quaternary LDPC codes of size *n* able to carry *k* informative quads.

Neither BY barcodes of sizes *N* = 7, 8 (see Table 3) nor Golay barcodes of size *N* = 12 could satisfy the operational constraint *p*_*u*_ ≤ 10^−8^ and *M* ≥ 24. Only BY barcodes of size *N* = 15 could satisfied it but at the expense of a remarkable increment in the bound *p*_*e*_ of the rate of read losses which approximates 10^−2^. Although Golay barcodes were able to improve BY barcodes by allowing *M* = 1545 with *p*_*e*_ = 8.1 10^−4^, they exhibited an inferior *p*_*u*_ performance—*p*_*u*_ = 2.4 10^−5^. Finally, among random barcodes, only those with *d*_*H*_ = 5 (see Table 4) were able to satisfy the operational constraint. Similarly to BY barcodes, this was accomplished at the expense of high rates of read losses, in the order of 10^−2^.

**Table 3:** The performance of BY barcodes.

				*p*_*s*_ = 10^−2^			
N	M	B	(n, k, t)	*p*_*e*_	$p_{e}^{+}$	*p*_*u*_	$p_{u}^{+}$
7	117	0.491	(7, 4, 1)	2.20 10^−3^	2.21 10^−3^	2.12 10^−4^	2.13 10^−4^
8	111	0.424	(8, 4, 1)	2.87 10^−3^	2.88 10^−3^	2.37 10^−6^	2.38 10^−6^
15	2880	0.383	(15, 11, 1)	9.94 10^−3^	9.95 10^−3^	0	2.00 10^−9^

BY barcodes of size *N* over a *QSC* model where mismatch errors occur with probability *p*_*s*_. *M*, *B*, *p*_*e*_ and *p*_*u*_ are respectively the empirical estimates of the multiplexing capacity, the barcoding rate, the probability of barcodes identification error and the probability of undetected multiplexing errors; $p_{e}^{+}$ and $p_{u}^{+}$ are the upper error bars of the two latter ones. Underlying codes are quaternary extensions of binary Hamming codes of size *n* able to carry *k* informative bits and to correct at least *t* binary errors.

**Table 4:** The performance of Random barcodes.

				*p*_*s*_ = 10^−2^			
N	*d*_*H*_	M	B	*p*_*e*_	$p_{e}^{+}$	*p*_*u*_	$p_{u}^{+}$
8	5	24	0.286	2.75 10^−2^	2.76 10^−2^	0	2.00 10^−9^
8	3	531	0.565	7.72 10^−2^	7.73 10^−2^	5.80 10^−7^	5.81 10^−7^
9	7	6	0.143	1.94 10^−3^	1.95 10^−3^	0	2.00 10^−9^
9	5	62	0.330	3.11 10^−2^	3.12 10^−2^	0	2.00 10^−9^
9	3	1936	0.606	8.64 10^−2^	8.65 10^−2^	8.80 10^−7^	8.82 10^−7^
10	7	13	0.185	2.42 10^−3^	2.43 10^−3^	0	2.00 10^−9^
10	5	164	0.367	3.47 10^−2^	3.48 10^−2^	1.01 10^−8^	1.02 10^−8^
10	3	7198	0.640	9.56 10^−2^	9.57 10^−2^	1.13 10^−6^	1.14 10^−6^

Random barcodes of size *N* with minimum edit distance [56] equal to their minimum Hamming distance *d*_*H*_ over a *QSC* model where mismatch errors occur with probability *p*_*s*_. *M*, *B*, *p*_*e*_ and *p*_*u*_ are respectively the empirical estimates of the multiplexing capacity, the barcoding rate, the probability of barcodes identification error and the probability of undetected multiplexing errors; $p_{e}^{+}$ and $p_{u}^{+}$ are the upper error bars of the two latter ones.

## Discussion

Simulation results suggest that regarding the design of critical barcoding systems for NGS platforms mainly impaired by mismatch errors, barcodes built from quaternary LDPC codes may perform better than those built from powerful binary BCH and Golay codes, pseudo-quaternary Hamming codes and random designs. As a result, careful planning of ubiquitous multiplex sequencing projects may be accomplished with LDPC barcodes. It may be argued that LDPC barcodes are two or three times larger than commercial random barcodes currently in use and systematic barcoding designs based on Hamming or Golay codes, which at most require a handy number of bases. In agreement with [57], our results suggest that there could be a high price to paid for using such small barcoding systems, either high rates of critical undetected multiplexation errors or high rates of read losses must be tolerated.

It may be also argued that BCH, or even Golay, barcoding performance may be improved with more sophisticated decoding algorithms, e.g., with those able to exploit the reliability of received symbols [58]. We note, however, that in the DNA barcoding framework, reliability information about received symbols is only available at the quaternary sequencing layer. Although reliability information of quaternary symbols might be easily exported to higher order fields, e.g., if pairs of DNA bases were packed into hexadecimal symbols of GF(16), it cannot be exported to lower order fields, e.g., to the binary level where actual demultiplexing of BCH or Golay barcodes takes place. In other words, mapping DNA bases to binary tuples by means of BCH or Golay codes implies the mandatory use of, probably suboptimal, binary algebraic decoding algorithms at the demultiplexing stage.

The promising performance of quaternary LDPC barcodes is built upon the introduction of a novel method for the selection of suitable sparse and short quaternary parity check matrices and the use of iterative decoding algorithms. The selection method subsumes convenient structural properties of general non-binary LDPC matrices into a simple score thus allowing the rapid generation of candidate LDPC barcodes involving few tens of bases. Candidate LDPC barcodes can then be checked for the satisfaction of a variety of sequencing chemical constraints. Since barcodes verification is expected to be easier than their *ab-initio* design, quaternary LDPC barcodes bring an affordable computational solution for the design of practical barcoding systems with stringent constraints on the probability of read losses and the probability of undetected multiplexation errors.

In this paper, LDPC barcodes demultiplexing was performed with the iterative decoding algorithm used for decoding non-binary LDPC codes for magnetic recording applications [59]. Hence, LDPC barcodes demultiplexing complexity scales with O (*m* × *q* × *j* × (*log*_2_ *q*+*j*)) per iteration, being *m* = *n* − *k* the number of redundant symbols of the regular non-binary LDPC in GF(q) and *j* the number of non-zero entries per column of the LDPC matrix [60, 61]. This demultiplexing complexity, which can be considered manageable up to *q* = 16 [62], is amenable for hardware implementation [63]. For example, for LDPC barcodes of size *N* = 19 allowing the multiplexation of up to *M* = 65 samples using a quaternary regular (*n* = 19, *k* = 4) LDPC code with *j* = 3, the recovery of 12M identities in an Illumina MiSeq platform with a 177 MIPS processor using a maximum of 50 iterative decoding steps would take less than an hour.

Along this paper we have restricted our attention to sequencing errors of the mismatch type. Readers might reasonable argue that challenging sequencing errors are those dominated by insertions and deletions. We note, however, that these errors might be also tackled with short *non*-binary LDPC codes. Specifically, concatenated watermark codes [64] built from an outer *non*-binary LDPC code and an inner sparse code, to which a watermark sequence has been added, could be used. Concatenated watermark codes rely on the ability of the inner decoder to transform insertion/deletion errors into mismatch errors and on the ability of the outer *non*-binary LDPC decoder to correct them. Regarding DNA barcoding applications of concatenated watermark codes, we expect samples identities are first mapped to hexadecimal strings, that these strings are LDPC encoded with a short hexadecimal LDPC code already optimized for transmissions over a quaternary symmetric channel, that hexadecimal LDPC codeword symbols are mapped to the quaternary sequencing layer with a non-linear quaternary sparse code and that resulting sparse quaternary sequences are finally perturbed with the addition of a well-known quaternary pseudorandom sequence defined as the pilot watermark signal. After this non-trivial processing of samples identities is performed, candidate barcode sequences able to deal with mismatch and indel sequencing errors could be obtained. We are currently putting together these pieces, looking forward to the design of concatenated watermark barcodes for the third generation Single-Molecule Real-Time (SMRT) long-read sequencing technology [65], for which indel and mismatch error rates may range up to 14 and 1% respectively [66]. We thus conclude that barcodes derived from generalized LDPC codes in GF(q) may be good candidates for improving the multiplexing capacity of current 2/3G and upcoming 4G [67] sequencing technologies.

## Methods

### LDPC codes in GF(q)

Let us start with a brief revision of LDPC codes in GF(q), *q* = 4^*u*^ and *u* ≥ 1. Let Γ be a bipartite graph with *n* left nodes called message nodes and *m* < *n* right nodes called check nodes. Let us map one-to-one the *n* message nodes of Γ to the *n* coordinates of *q* − *ary* codewords **c** = (*c*_1_,…,*c*_*n*_), *c*_*i*_ ∈ {0, 1, 2, …,*q*−1}, *i* = 1,…,*n*. Provided the sum of neighboring positions for all check nodes among neighboring message nodes is zero in GF(q), Γ defines a *q-ary* linear code of size *n* able to carry *k* = *n* − *m* informative symbols. Thus, the code structure can be dissected into *m* component parity subcodes. In addition, if Γ is sparse, i.e., each message node is constrained by *j* < < *m* check nodes and each check node constraints *v* < < *n* message nodes, the code turns to be an LDPC code in GF(q). Finally, if *n* ⋅ *j* = *m* ⋅ *v* holds, a regular LDPC code is obtained.

**Fig 3:**
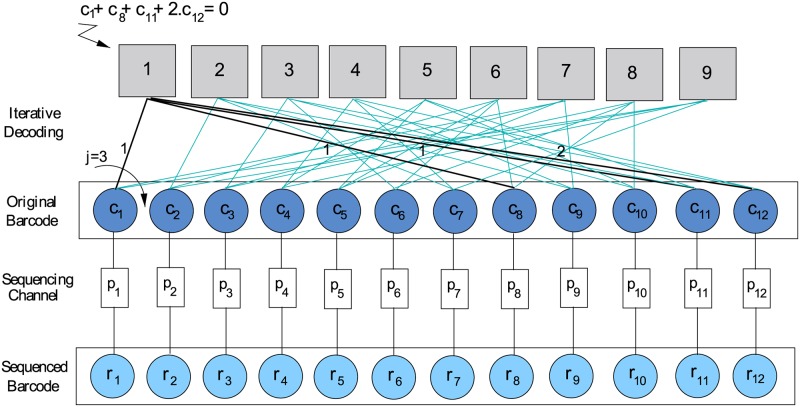
The factor graph of an LDPC barcoding system built from a 4-ary LDPC code of size *n* = 12 able to carry *k* = 3 informative quads and thus, to induce 64 candidate barcode sequences. Each codeword symbol *c*_*i*_, *i* = 1, …, 12, is constrained by exactly *j* = 3 parity subcodes. The LDPC code is built from *m* = 9 parity subcodes, e.g., *c*_1_ + *c*_8_ + *c*_11_ + 2 *c*_12_ = 0 holds. A *QSC* generates mismatch sequencing errors with probabilities *p*_*i*_ = *p*_*s*_ and thus, corrupted barcode bases *r*_*i*_ are observed after sequencing, *i* = 1, …, 12. At *p*_*s*_ = 0.01 this system can multiplex up to *M* = 15 samples with *p*_*e*_ = 10^−4^ and *p*_*u*_ ≈ 0.

LDPC codes can be depicted by means of factor graphs [68]. Circles are used to represent original codeword symbols *c*_*i*_ and their noisy observations *r*_*i*_, *i* = 1, …, *n*. Rectangles are used to represent parity constraints over codeword symbols. Edges are put between codeword symbols and parity constraints. Legal LDPC codewords must fulfill the complete set of *m* parity constraints. On regular LDPC codes, any codeword symbol participates in exactly *j* ≥ 3 parity constraints. Rectangles are also used to represent probability functions *p*_*i*_(*a*, *b*) = *P*(*r*_*i*_ = *a*∣*c*_*i*_ = *b*) modelling the transmission channel, *i* = 1…*n*. For a QSC model, *p*_*i*_(*a*, *a*) = 1−*p* and $p_{i}\left(b,a\right)=\frac{p}{3}$ for *a* ≠ *b* holds. Iterative decoding provides estimates ${\hat{c}}_{i}$ given *p* and *r*_*i*_, *i* = 1,…,*n* (see Fig 3).

The construction of barcodes from quaternary LDPC codes is straightforward once Γ is given. Formally, Γ is described by the so-called parity check matrix **H**, an sparse matrix with *m* rows and *n* columns conveying *j* < < *m* non-zero entries per column and *v* non-zero entries per row, *j* ⋅ *n* = *v* ⋅ *m*. For quaternary LDPC codes, non zero-entries in **H** are taken from the set {1, 2, 3}. From **H**, the so-called generator matrix **G** with *k* = *n* − *m* rows and *n* columns can be obtained. Practically, **G** allows the straightforward generation of LDPC codewords **c** from message vectors *s* = (*s*_1_, …, *s*_*k*_), *s*_*i*_ ∈ {0, 1, 2, 3}, *i* = 1,…,*k*, by means of **c** = **s** ⋅ **G**.

### Good short quaternary LDPC codes

To shed light into the ability of the *D* score to discriminate between prospective good and bad short quaternary LDPC codes, 20 sets of 50 random quaternary LDPC matrices were built from binary PEG templates with *m* rows, *n* columns and *j* = 3 non-zero entries per column. For each set, the best *D*-scoring code was selected and its barcoding performance assessed at *p*_*s*_ = 10^−2^. The Spearman correlation coefficient *S* ^2^ was then used to evaluate the correlation between *D* scores and the empirical *log*_10_ *p*_*e*_ accomplished by selected LDPC codes. Moderate negative associations were observed suggesting that the *D* score was indeed useful for the identification of prospective good short quaternary LDPC codes for barcoding purposes. For example, the observed correlation at *p*_*s*_ = 10^−2^ for LDPC barcodes of size *N* = 24 carrying *k* = 5 informative quads was *S* ^2^ = −0.52, *p*-value < 0.05. Practically, prospective good short quaternary LDPC codes were selected from sets of 1000 random instances. For each selected **H**, the corresponding generator matrix **G** with *k* rows and *n* columns was computed using the constraint **G** ⋅ **H** ^*t*^ = **0**. This was accomplished by means of an adaptation of I.V. Kozintsev software [69] for the inversion of **H** matrices in *GF*(*q*) using Gaussian elimination.

### Estimation of *p*_*e*_ and *p*_*u*_

Simulation experiments were performed to analyze the robustness of BCH, Golay, LDPC, BY and Random barcodes over the *QSC* model. Corrupted BCH barcodes were first deinterleaved, mapped to the binary domain using inverse mapping tables and decoded with the implementation of the Berlekamp-Massey decoding algorithm in [70]. Decoded codewords in *GF*(2) were then mapped to *GF*(4) to recover original barcode sequences. A similar procedure was used to recover corrupted Golay, LDPC and BY barcodes. Golay barcodes were first deinterleaved, mapped to the binary domain and then decoded with the implementation of the arithmetic decoding algorithm in [71]. LDPC barcodes were first deinterleaved and then decoded with the iterative decoding algorithm for quaternary LDPC codes described in [29] and implemented in [72]. The LDPC decoding algorithm was set to work with a maximum of 50 iterations with the probability *p*_*s*_ of a base mismatch used as input to the *QSC* channel model. BY barcodes were first deinterleaved and then decoded as indicated in [27]. Finally, random barcodes taken from [56] with experimentally determined minimum Hamming distance were simply decoded with a bounded distance decoder.

The probability *p*_*e*_ of barcode identification error was then estimated by Montecarlo simulation. For this purpose, *T* = 100 random samples comprising *C* = 10^7^ barcode sequences were used. Samples were obtained by performing sampling with replacement over the sets of valid barcode sequences obtained after the Barcrawl filtering stage. For each sample *S*_*i*_, the proportion *p*_*e*,*i*_ of barcodes identification errors and sample variances $s^{2_{i}}=\frac{1}{C}\times p_{e,i}\left(1-p_{e,i}\right)$ were computed, *i* = 1, …, *T*. At the end, the pooled sample mean ${\bar{p}}_{e}=\left(\sum_{i}p_{e,i}\right)/T$, the pooled sample standard deviation $s_{p}=\sqrt{\left(\sum_{i}s_{i}^{2}\right)/T}$ and the pooled standard error $se_{p}=s_{p}\sqrt{T/C}$ were computed. If ${\bar{p}}_{e}\neq 0$, then 95% confidence intervals $\left[{\bar{p}}_{e-},{\bar{p}}_{e+}\right]$ were computed as ${\bar{p}}_{e}\pm 2\times se_{p}$. On the other hand, if ${\bar{p}}_{e}=0$, then ${\bar{p}}_{e-}=0$ and ${\bar{p}}_{e+}=1-exp\left(-2/CT\right)$ were used [41]. A base 10 logarithmic scale was used to graphically report *p*_*e*_ estimations and thus, 95% confidence intervals for the case ${\bar{p}}_{e}\neq 0$ were graphically reported as $log_{10}\left({\bar{p}}_{e}\right)\pm 2\times 0.434log_{10}\frac{se_{p}}{{\bar{p}}_{e}}$[73]. A similar procedure was used for estimating the probability *p*_*u*_ of undetected multiplexation errors.

### Estimation of *M* and *B*

The multiplexing capacity *M* and the barcoding rate *B* are fundamental properties of any coding-based barcoding system. They follow from counting all candidate barcode sequences that are compatible with a predefined set of DNA manipulation constraints. For random barcodes of size *N*, 4^*N*^ candidate barcode sequences must be first individually screened to remove those with undesirable composition patterns. Concerning their posterior use for error correction purposes, remaining sequences must be then globally analyzed to ensure a predefined minimum distance. Hence, for random barcodes, the estimation of *M* and *B* scales exponentially with *N* both in time and memory. On the other hand, the minimum distance is a built-in property of candidate barcode sequences when systematic error correcting codes are used and thus, only the presence of undesirable composition patterns must be controlled. For barcodes of size *N* built from linear codes of size *n* able to carry *k* informative symbols in GF(q), just *q* ^*k*^ sequences must be individually screened, *k* < < *n*. For modest *k* and *q* settings, e.g., *k* < 8 for *q* = 4, exact estimation of *M* and *B* can be accomplished. For larger *k* settings, a Montecarlo approach is required.
